# Easy Method of Preparing Iodide of Potassium

**Published:** 1847-06

**Authors:** 


*Easy Method of preparing Iodide of Potassium.*—M. Pypers subjects to a moderate heat a mixture of 100 parts of iodine, 75 of carbonate of potash, 30 of iron filings, and 120 of water. The mass is to be dried and then heated to redness; the resulting reddish powder is to be treated with water, and the solution obtained filtered and evaporated to dryness. One hundred parts of iodine yield 135 of very white but slightly alkaline iodide of potassium.—*Ibid, from Journ. de Chim. Med.*

